# Eicosapentaenoic Acid Is Associated with Decreased Incidence of Alzheimer’s Dementia in the Oldest Old

**DOI:** 10.3390/nu13020461

**Published:** 2021-01-30

**Authors:** Debora Melo van Lent, Sarah Egert, Steffen Wolfsgruber, Luca Kleineidam, Leonie Weinhold, Holger Wagner-Thelen, Wolfgang Maier, Frank Jessen, Alfredo Ramirez, Matthias Schmid, Martin Scherer, Steffi G. Riedel-Heller, Michael Wagner

**Affiliations:** 1 German Center for Neurodegenerative Diseases (DZNE), 53127 Bonn, Germany; Steffen.Wolfsgruber@dzne.de (S.W.); frank.jessen@uk-koeln.de (F.J.); Alfredo.Ramirez@uk-koeln.de (A.R.); matthias.c.schmid@uni-bonn.de (M.S.); Michael.Wagner@dzne.de (M.W.); 2 Glenn Biggs Institute for Alzheimer’s and Neurodegenerative Diseases, University of Texas Health Science Center, San Antonio, TX 78229, USA; 3 Institute of Nutritional Medicine, University of Hohenheim, 70599 Stuttgart, Germany; sarah.egert@uni-hohenheim.de; 4 Department of Neurodegenerative Diseases and Geriatric Psychiatry, University Hospital Bonn, 53127 Bonn, Germany; Luca.Kleineidam@ukbonn.de (L.K.); holger.wagner-thelen@uk-koeln.de (H.W.-T.); wolfgang.maier@ukb.uni-bonn.de (W.M.); 5 Department of Medical Biometry, Informatics and Epidemiology, University Hospital Bonn, 53105 Bonn, Germany; weinhold@imbie.meb.uni-bonn.de; 6 Department of Psychiatry, Medical Faculty, University of Cologne, 50924 Cologne, Germany; 7 Division of Neurogenetics and Molecular Psychiatry, Department of Psychiatry and Psychotherapy, University of Cologne, Medical Faculty, 50937 Cologne, Germany; 8 Department of Primary Medical Care, Center for Psychosocial Medicine, University Medical Center, Hamburg-Eppendorf, 20146 Hamburg, Germany; m.scherer@uke.de; 9 Institute of Social Medicine, Occupational Health and Public Health, University of Leipzig, 04103 Leipzig, Germany; Steffi.Riedel-Heller@medizin.uni-leipzig.de

**Keywords:** fatty acids, eicosapentaenoic acid, omega-3, apolipoprotein E ε4, dementia, Alzheimer’s disease dementia, oldest old

## Abstract

Background. Omega-3 (n-3) and omega-6 (n-6) polyunsaturated fatty acids (PUFAs) may have different effects on cognitive health due to their anti- or pro-inflammatory properties. Methods. We aimed to prospectively examine the relationships between n-3 and n-6 PUFA contents in serum phospholipids with incident all-cause dementia and Alzheimer’s disease dementia (AD). We included 1264 non-demented participants aged 84 ± 3 years from the German Study on Ageing, Cognition, and Dementia in Primary Care Patients (AgeCoDe) multicenter-cohort study. We investigated whether fatty acid concentrations in serum phospholipids, especially eicosapentaenoic acid (EPA), docosahexaenoic acid (DHA), alpha-linolenic acid (ALA), linoleic acid (LA), dihomo-γ-linolenic acid (DGLA), and arachidonic acid (AA), were associated with risk of incident all-cause dementia and AD. Results. During the follow-up window of seven years, 233 participants developed dementia. Higher concentrations of EPA were associated with a lower incidence of AD (hazard ratio (HR) 0.76 (95% CI 0.63; 0.93)). We also observed that higher concentrations of EPA were associated with a decreased risk for all-cause dementia (HR 0.76 (95% CI 0.61; 0.94)) and AD (HR 0.66 (95% CI 0.51; 0.85)) among apolipoprotein E ε4 (APOE ε4) non-carriers but not among APOE ε4 carriers. No other fatty acids were significantly associated with AD or dementia. Conclusions. Higher concentrations of EPA were associated with a lower risk of incident AD. This further supports a beneficial role of n-3 PUFAs for cognitive health in old age.

## 1. Introduction

The number of elderly and oldest-old (85+ years) people around the world is rising, and so is the number of individuals with dementia [1,2]. Dementia is characterized by deficits in one or more cognitive domain, such as aphasia or executive dysfunction [3]. The primary kind of dementia is Alzheimer’s disease (AD) dementia. It has been hypothesized that the accumulation of protein beta-amyloid ((Aβ) plaques) and the accumulation of the protein tau (tau tangles) in the brain might contribute to the damage of neurons causing AD dementia [4,5]. Our food habits throughout the lifespan may have a predominant role in modulating our personal risk to dementia, either directly or through modulation of dementia risk factors, such as diabetes mellitus type 2 and cardiovascular diseases [6,7,8].

Regular consumption of fatty fish, for example, has been associated with slower cognitive decline [9]. In addition, previous longitudinal studies have reported relationships between higher blood concentrations of polyunsaturated fatty acids (PUFAs) and decreased risk of developing dementia [10,11]. These protective effects have been primarily ascribed to the long chain omega-3 (n-3) PUFA, eicosapentaenoic acid (EPA), and docosahexaenoic acid (DHA), which are present in fatty fish [12]. Both fatty acids function as anti-inflammatory and neuronal protective agents in the brain, which may protect against dementia [11]. The role of their plant-derived precursor alpha-linolenic acid (ALA) is less clear, although it is the principle dietary n-3 PUFA consumed in the typical Western diet [12]. In contrast, n-6 PUFAs (especially arachidonic acid) contribute to the production of inflammatory agents, which are risk factors for dementia [13].

In addition, it has been hypothesized that the apolipoprotein ε4 (APOE-ε4) allele is the major genetic risk factor for the most common form of dementia; AD dementia has an influence on lipid homeostasis deregulation [14,15]. Previous observational studies have examined the interaction between PUFA (intake) and APOE ε4. One study has found an interaction between n-3 PUFA intake and APOE ε4 when investigating cognitive decline [16]. The authors reported an association between moderate to high intake of n-3 PUFA and slower rates of cognitive decline among APOE ε4 carriers, but not among APOE ε4 non-carriers [16]. Other studies, however, have failed to find an interaction between blood concentrations of fatty acids and APOE ε4 status [17,18]. Therefore, longitudinal studies, such as the presented study, are needed to elucidate this further.

We aimed to prospectively examine the relationships between n-3 and n-6 PUFA contents in serum phospholipids with incident all-cause dementia and AD dementia in the oldest old. Recently, the oldest old (85+ years) have received great interest in the dementia field in general. However, not much is known with regard to the relationship between nutrition and neurodegenerative diseases in this rapidly increasing age group afflicted with a high incidence of dementia.

## 2. Materials and Methods

### 2.1. AgeCoDe Study Design and Participants

The current data are from the German Study on Ageing, Cognition and Dementia in Primary Care Patients (AgeCoDe) and Needs, health service use, costs and health-related quality of life in a large sample of oldest-old primary care patients (AgeQualiDe). The study is a German multicenter and general practitioner (GP) registry-based prospective cohort study on early detection and prediction of mild cognitive impairment and dementia in elderly primary care patients starting in 2003. Enrolled participants were dementia-free primary care patients age 75 years or older, who had at least one personal contact with their GP during the past year, and who were living in the urban areas of the six German cities: Bonn, Düsseldorf, Hamburg, Leipzig, Mannheim, or Munich [19]. The recruitment was conducted by 138 GPs connected to the respective study sites. Baseline visits were conducted between January 2001 and November 2003. Since then, eight follow-up (FU) visits (with an 18-month interval between each FU) were completed up to the time of the present study. Selection and sampling of the participants have been described previously [19,20]. At baseline, 3327 participants were successfully contacted and consented to enrollment in the study. For participants who could not be interviewed personally at FU visits, informant-based information was obtained. In such a case, participants were excluded from further FUs. AgeCoDe assessments were performed by trained investigators (physicians, psychologists, and gerontologists) at participants’ homes and included structured clinical interviews comprising clinical, sociodemographic, and anthropometric information; neuropsychological tests; current physical and mental health; and psychosocial and lifestyle factors. The study was approved by the local ethics committees of the six participating centers, and all participants gave their written informed consent to the study.

### 2.2. Study Participants

**Figure 1:**
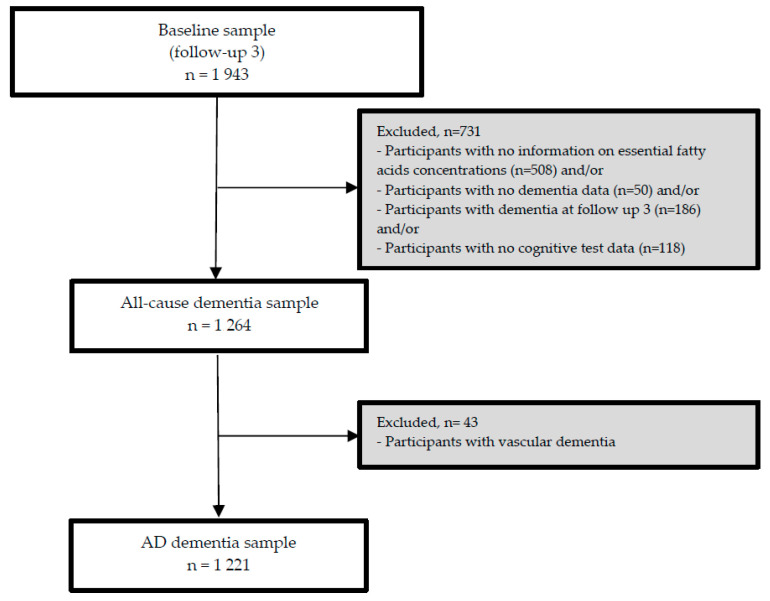
Flow chart of the participants included in the study.

The third follow-up visit (FU-3) forms the analytical baseline of the present study. As the nutrient biomarker data were available at FU-3, we used data from participants who attended FU-3 and at least one of the additional FU visits (until FU-8). From 1943 participants at FU-3, we excluded participants with any dementia at or before FU-3 (*n* = 186) and/or those for whom serum concentrations of PUFA at FU-3 (*n* = 508) and/or follow-up data on dementia (*n* = 50) and/or cognitive test data (*n* = 118) were not available at subsequent FU visits (*n* = 731). Consequently, the sample for our longitudinal analyses included 1264 participants (incident all-cause dementia sample), and 1221 in the AD dementia subsample (with incident AD dementia or without any dementia, during FU, excluding 43 incident non-AD dementia cases). Details on the exclusion of participants of the present study are presented in Figure 1.

### 2.3. Fatty Acids

#### 2.3.1. Processing and Analysis

Blood was collected by participants’ GPs in tubes with EDTA and without anticoagulant and stored at −80 °C. The fatty acid composition of serum phospholipids was determined in duplicate by gas chromatography (Shimadzu GmbH, model GC 2010 plus, Duisburg, Germany, flame ionization detector [FID]) as described previously [21,22]. Briefly, serum samples (1 mL) were defrosted at room temperature, and an internal standard (C15; 1,2-dipentadecanoyl-sn-glycero-3-phosphatidylcholine; Larodan, Monroe, MI, USA) was added. Total lipids were extracted using methanol:chloroform (1:2, v:v) [22] according to a modified Folch method [23].The phospholipid fraction was separated using thin-layer chromatography according to the method of Christophe and Matthijs (1967) using a silica thin-layer chromatography plate (phase SIL G UV254; layer thickness 0.25 mm; 5 × 10 cm^2^; Macherey-Nagel GmbH & Co. KG, Düren, Germany) in a solvent mixture of petroleum ether and acetic acid (17:3 by volume) [21,24]. After scraping off the phospholipid band under ultraviolet light, the phospholipid fraction was methylated by transesterification with methanol/HCl (25:1 by volume) and incubated at 95 °C for 4 h. The fatty acids methyl esters were extracted with petroleum ether, dissolved in heptane, and injected into the gas chromatograph. Helium was used as carrier gas with a constant flow of 4 mL/min. Injector and flame ionization detector temperatures were 200 °C and 250 °C, respectively [22]. Peaks of interest were identified by comparing with authentic fatty acid methyl ester standards (37 Component FAME Mix certified reference material, C14–C24, Sigma-Aldrich, St. Louis, MI, USA). Selected fatty acids were expressed as absolute concentration and as a percentage of the total area by dividing the integrated area under the peak by the total area of all fatty acids. Fatty acids were also determined quantitatively from the internal standard (see above) and expressed as µmol/L serum.

#### 2.3.2. Fatty Acids as Concentration and Percentage Distribution

Blood contents of PUFA have been widely used as biomarkers of intake and surrogates of their enrichment in cellular membranes [25]. Herein, studies frequently reported contents of these fatty acids as (relative) percentage distribution of total fatty acids in a given compartment (e.g., serum, serum phospholipids, or cells) instead of absolute fatty acid concentration [26], as percentage distribution tends to have a lower variability compared to absolute concentration [27]. However, the advantage of absolute concentration is that the fatty acid levels do not depend on the levels of other fatty acids. Brenna et al. (2018) recommend to report both concentration and percentage distribution [27], because previous research has shown that the relationship between fatty acids changes depending on whether they are expressed as percentage or as concentration [24]. Therefore, we report PUFAs as both concentration and percentage distribution in the present study.

### 2.4. Dementia

Dementia was diagnosed by consensus of the interviewing investigator and an experienced geriatrician or geriatric psychiatrist according to the Diagnostic and Statistical Manual of Mental Disorders 4th Edition (DSM-IV) and International Classification of Diseases (ICD-10) criteria that are implemented as a diagnostic algorithm in the Structured Interview for Diagnosis of Dementia of Alzheimer type, Multi-infarct Dementia and Dementia of other Aetiology according to DSM-IV and ICD-10 (SIDAM) [28,29]. This algorithm comprises cognitive impairment, as defined by the total SIDAM cognitive score (SIDAM score (SISCO)), scoring 0–55, with a higher score indicating a better performance than the sum of the Mini-Mental State Examination (MMSE) score (0–30) and 25 additional items) and impairment of activities of daily living (ADL) as defined by a score of at least two points on the SIDAM-ADL-scale. For dementia, the etiological diagnosis of AD dementia was established according to the National Institute of Neurological and Communicative Disorders and Stroke and the Alzheimer’s Disease and Related Disorders Association (NINCDS-ADRDA) criteria for probable AD dementia [30]. For the diagnosis of vascular dementia, that is, in cases of evidence for cerebrovascular events (Hachinski–Rosen scale, medical history) and a temporal relationship between the cerebrovascular event and the occurrence of cognitive decline, the National Institute of Neurological Disorders and Stroke and Association Internationale pour la Recherché et l’Enseignement en Neurosciences (NINDS-AIREN) criteria were used [31]. Mixed dementia was diagnosed in cases of cerebrovascular events without temporal relationship to cognitive decline. Dementia diagnosis in participants who were not personally interviewed was based on the Global Deterioration Scale (GDS) [32] and the Blessed Dementia Rating (BDR) scale [33]. A score of four or higher on the GDS was used as the criterion for the dementia diagnosis. In these cases, an etiological diagnosis was established if the information provided was sufficient to judge etiology according to the above-named criteria. In addition, for statistical analyses, AD dementia and mixed dementia were combined into one AD dementia group.

### 2.5. Confounders

Assessments were performed by trained investigators (physicians, psychologists, and gerontologists) at participants’ homes and included structured clinical interviews comprising clinical, sociodemographic and anthropometric information; neuropsychological tests; current physical and mental health; and psychosocial and lifestyle factors.

Education was classified into three categories (low, middle, and high) based on the Comparative Analysis of Social Mobility in Industrial Nations (CASMIN) classification system [34]. APOE genotyping was performed according to standard procedures [35]. Participants were grouped into those with at least one APOE ε4 allele (homo- and heterozygous carriers; positive APOE ε4 status) and those without an ε4 allele (noncarriers, negative APOE ε4 status). Body height and weight were measured at FU-3. BMI was calculated based on weight (kg) divided by height squared (m^2^). Smoking status was assessed at the AgeCoDe baseline visit and used as a proxy for the present study (FU-3). Smoking status was categorized into three categories: never smoker, former smoker, and current smoker. Assessment of physical activity was conducted at FU-3. We constructed a modified physical activity score based on Verghese et al. [36]. In brief, participants reported the frequency of usual engagement in each of the six physical activities: bicycling, walking, swimming, gymnastics, chores/gardening, and a category of other physical leisure activities (e.g., bowling, jogging, or golfing), using five possible answers: (1) “each day”; (2) “several times per week”; (3) “once a week”; (4) “less than once a week”; and (5) “never”. For the present study, the five frequency categories were collapsed into two categories whether the participant usually engaged in one of the six activities (once a week to each day = 1) or not (less than once a week or never = 0). For each participant, these values (0 or 1) were summed up across the six activities to a total physical activity score (score: 0–6). Serum concentration of vitamin E (alpha-tocopherol) was determined by high-performance liquid chromatography (HPLC) and ultraviolet detection as described previously [37]. Serum concentration of total cholesterol (FU-3) was determined using polychromatic endpoint measurement, and serum concentration of triglycerides (FU-3), was determined using biochromatic endpoint measurement with a Dimension Vista 1500 analyzer (Siemens Healthcare Diagnostics GmbH) [37]. Cognitive decline was calculated using the Consortium to Establish a Registry for Alzheimer’s Disease (CERAD) neuropsychological assessment battery (we applied the validated German version [38]), a 10-item Word List Delayed Recall subtest (scoring 0–10; higher scores indicating a better memory performance). We subtracted the Delayed Recall performance at FU-3 from the Delayed Recall performance at the AgeCoDe baseline in order to derive a measure of cognitive decline prior to FU3. Lastly, lipid-lowering medication (i.e., statins and fibrates) was categorized into users and non-users.

### 2.6. Statistical Analysis

In the present study, we investigated PUFAs as absolute concentrations and relative fatty acid profiles in serum phospholipids: alpha-linolenic acid (C18:3 n-3; ALA), eicosapentaenoic acid (C20:5 n-3; EPA), docosahexaenoic acid (C22:6 n-3; DHA), linoleic acid (C18:2 n-6; LA), dihomo-γ-linolenic acid (C20:3 n-6; DGLA), and arachidonic acid (C20:4 n-6; AA).

Cox proportional hazard models were used to investigate the longitudinal associations between the effect of one standard deviation (SD) increase in n-3 and n-6 PUFAs and incidence of AD dementia or all-cause dementia. We tested the proportional hazards assumption using the cox.zph function of the statistics software R. No violations of the proportional hazards assumption were detected.

Confounders were selected based on published literature. Six confounders (BMI, APOE ε4, vitamin E, total cholesterol, triglycerides, and physical activity) contained missing values (Table S1). The percentages of missing values ranged from 2.1% (physical activity) to 19.9% (triglycerides) in the longitudinal analysis. To account for potential attrition bias, multiple imputation was used by creating ten different possible copies of the original dataset, in which the missing values were substituted by imputed values (Table S1) [39]. A description of the procedure is reported in Table S8.

Models were adjusted for sociodemographic factors: age, gender, education and APOE ε4 status (Model 1); in addition to Model 1, we adjusted for the lifestyle factors BMI, smoking status and physical activity, vitamin E, total cholesterol, triglycerides, lipid-lowering medication, and cognitive decline before FU-3 (Model 2). Inclusion of this cognitive decline measure as a covariate is one way to account for reverse causality, which might be present if incipient dementia (reflected in ongoing cognitive decline) changes dietary habits and fatty acid levels even before a diagnosis is made. We investigated interactions between APOE ε4 status and the n-3 and n-6 PUFAs when investigating AD dementia and all-cause dementia in Model 2. When P_interaction_ (<0.10) was significant, stratified analyses were performed according to APOE ε4 status (carrier or non-carrier).

*p* < 0.05 was considered statistically significant. IBM SPSS Statistics for Windows (Release 23) was used to perform the analyses.

## 3. Results

Table 1 details the study population characteristics. The mean age of the sample was 84 (standard deviation = 3) years, females were slightly more represented than males, and the mean BMI was 26 (standard deviation = 3.8) kg/m^2^. The APOE ε4 allele was present in 19.0% of the participants. Mean concentrations of ALA, EPA, DHA LA, DGLA, and AA are reported in Table 2 together with Spearman correlation coefficient for each fatty acid measured as concentration and as proportion (range between 0.545 (n-6: AA) and 0.861 (n-3: EPA). Over approximately seven years of FU, 233 participants developed all-cause dementia, including 190 participants who developed AD dementia.

### 3.1. Associations between Concentrations of n-3 and n-6 PUFAs and Incident All-Cause Dementia and Incident AD Dementia

Higher serum phospholipid EPA concentrations were borderline significantly associated with a lower incidence of all-cause dementia (Model 1: hazard ratio (HR) = 0.86, 95% CI 0.74, 1.01) (Table 3). After additional adjustments for Model 2 confounders the association disappeared. We observed no significant associations between the other PUFAs and incident all-cause dementia. However, higher EPA concentrations was significantly associated with a lower incidence of AD dementia in both models (Model 2: HR = 0.76, 95% CI 0.63, 0.93) (Table 4). Moreover, higher DHA concentrations were associated with a lower incidence of AD dementia in model 1 (HR = 0.84, 95% CI 0.71, 0.99), but the association did not remain in Model 2 (HR = 0.87, 95% CI 0.74, 1.04). We observed no significant associations between the other PUFAs and incident AD dementia. In addition, no significant relationships were observed between all investigated PUFAs and incident vascular dementia (data not shown). Furthermore, in addition to Model 1 and 2 covariates, we adjusted the associations under study for depression, diabetes, coronary heart disease, and heart failure. These additional adjustments did not change the results (data not shown).

### 3.2. Associations between Proportions of n-3 and n-6 PUFAs and Incident All-Cause Dementia and Incident AD Dementia

Higher proportions of EPA were borderline significantly associated with a lower incidence of AD dementia (Model 1: HR = 0.84, 95% CI 0.70, 1.00), but the association was no longer significant after additional adjustments for lifestyle factors, vitamin E, total cholesterol, triglycerides, cognitive decline, and lipid-lowering medication (Model 2: HR = 0.86, 95% CI 0.72, 1.03). No such associations were observed between the other n-3 and n-6 fatty acids and AD dementia or all-cause dementia (Table 5 and Table 6). Additional adjustments for depression, diabetes, coronary heart disease, and heart failure did not change the results (data not shown).

### 3.3. Secondary Analyses

We observed significant interactions between APOE ε4 and AA concentrations, APOE ε4 and ALA concentrations, APOE ε4 and EPA concentrations, and proportions when investing all-cause dementia, and APOE ε4 and EPA concentrations and proportions when investigating AD dementia (Tables S2–S7).

The relationship between higher concentrations of EPA and a decreased risk for incident all-cause dementia was significant among APOE ε4 noncarriers (Model 2: HR = 0.76, 95% CI 0.61, 0.94) but not among APOE ε4 carriers (Table S4). Furthermore, higher concentrations and higher proportions of EPA were associated with a decreased risk for incident AD dementia among APOE ε4 noncarriers (concentrations, Model 2: HR = 0.66, 95% CI 0.51, 0.84; proportions, Model 2: HR = 0.80, 95% CI 0.65, 0.99) but not among APOE ε4 carriers (Tables S5 and S7). Furthermore, we observed that the relationships between concentrations of AA, ALA, and proportions of EPA and incidence all-cause dementia among APOE ε4 carriers and non-carriers went mostly in the same direction as the main analyses, but the relationships were not significant (Tables S2, S3, and S6).

## 4. Discussion

In the AgeCoDe prospective multicenter cohort study, we investigated the prospective associations between concentrations and relative proportions of serum phospholipid n-3 and n-6 PUFAs and incident all-cause and AD dementia in the oldest old. We observed a relationship between higher EPA concentrations and a decreased incidence of AD dementia in the oldest old in fully adjusted models. Associations with DHA were attenuated and were no longer significant after multivariable adjustment. In addition, stratified analyses for APOE ε4 status revealed that higher concentrations of EPA were associated with a decreased incidence of all-cause dementia and AD dementia among APOE ε4 non-carriers but not among APOE ε4 carriers.

### 4.1. PUFA

#### 4.1.1. N-3 PUFA

Our n-3 PUFA findings are generally in line with previous prospective observational studies that measured fatty acids from blood [10,17,18,40,41,42]. The Three-City (3C) study in France, analyzing a younger sample with shorter follow-up and fewer incident cases, also observed an association between high relative proportions of plasma EPA and a decreased risk of developing all-cause dementia, while we observed a significant protective relationship with AD dementia [18] and a trend for EPA for all-cause dementia after full adjustment. Similar to our findings, the authors did not observe associations between higher proportion distributions of ALA and DHA and incident all-cause dementia in adjusted models [18]. A recent publication, however, from the same group reported a relationship between higher proportion distribution of DHA and dementia over a median FU time of 9.8 years [42]. The sample of the second study was slightly larger, which suggests that power might have been the reason for the null finding in the first 3C study and our study. In contrast to our study, the Women’s Health Initiative Memory Study reported no relationship between EPA and probable all-cause dementia [40]. In addition, the Canadian Study of Health and Aging found no association between high concentrations of total n-3 PUFA, DHA, or EPA measured in erythrocytes and all-cause and AD dementia [17]. Additionally our null findings for DHA are supported by the Framingham Heart study, which also reported no association between percentage plasma DHA and incident all-cause dementia [41]. In contrast to our and the single observational cohort studies, a meta-analysis (*n* = 25,872) of metabolites associated with general cognitive function reported an association between high DHA and lower risks of incident all-cause and AD dementia [10]. Thus, the overall evidence of observational studies today suggests that EPA and DHA may both counteract dementia, with discrepancies between studies resulting from limited statistical power and sample heterogeneity. Future studies are encouraged to collaborate and pool data together, as, to date, conflicting results are still being reported in intervention studies investigating the effect of n-3 PUFA on cognitive health. While some studies did find beneficial effects of total n-3 PUFAs or EPA and DHA separately [43,44,45,46,47,48,49,50], others did not [51,52,53,54,55,56,57,58,59], and only a few intervention studies have included or have been conducted among the oldest old [47,52,57,58,59].

#### 4.1.2. N-3 PUFA Mechanisms

Several potential underlying mechanisms may explain the investigated relationships. AD dementia is characterized by the accumulation of neurotoxic amyloid-beta (Aβ) peptide in the brain [11]. DHA and EPA, both present in the brain, are involved in the clearance of accumulated Aβ through insulin-degrading enzyme (IDE) [14]. In addition, PUFA concentrations have been associated with an increase in the non-amyloidogenic processing of amyloid precursor protein (APP), leading to an increased α-secretase-cleaved soluble APP (sAPPα) secretion, which has a neurotrophic and neuroprotective function [11]. Moreover, both PUFAs have anti-inflammatory properties, which are used to diminish inflammation in the brain, resulting in an enhancement of Aβ degradation [14]. Further, our effect differences of EPA after stratification for APOE ε4 status might be explained by the hypothesis that the APOE ε4 allele has an influence on lipid homeostasis deregulation [14,15]. It has been postulated that APOE ε4 contributes to the inhibition of lipid metabolism, inducing inefficient delivery of PUFAs to neurons in the brain and altered lipid membrane homeostasis [14,15,60].

#### 4.1.3. N-6 PUFA

The limited number of studies that investigate the relationships between n-6 PUFA and dementia have reported mixed results [18,61,62,63]. Similar to our study, the 3Ccohort study reported no associations between high proportions of LA or AA and incident all-cause dementia [18]. The Rotterdam study also reported no association between high dietary intake of LA and AD dementia [62] In contrast, an unexpected finding was published by the Chicago Health and Aging Project, which reported an association between high dietary intake of n-6 PUFA (i.e., including LA and AA) and a reduced risk for AD dementia after 3.9 years of FU [61]. Not many studies investigated DGLA. A single case–control study reported higher proportions of erythrocyte DGLA and AA in mild cognitively impaired participants in comparison to controls [63].

#### 4.1.4. N-6 PUFA Mechanisms

In contrast to n-3 PUFAs, n-6 PUFAs have pro-inflammatory properties. The predominant n-6 fatty acid is AA, which is converted to lipoxygenase or cyclooxygenase products [13]. These products have a pro-inflammatory, pro-atherogenic, and pro-thrombotic functions [13], which n-3 PUFAs fight against. As the biological changes of dementia start decades before dementia onset, future studies are encouraged to investigate the relationship between PUFA and preclinical endophenotypes of dementia, such as magnetic resonance imaging markers of brain aging and cerebral small vessel disease.

### 4.2. Measurement Methods of Fatty Acids

Our study shows that caution is needed for the interpretation of proportions of fatty acids, as the proportion of one fatty acid depends on the proportion of the other fatty acids [64]. As a result, when the concentration of one fatty acid is low, it does not necessarily mean a metabolic interaction (i.e., between fatty acid interaction) has taken place [64]. This might explain why we observed an association between absolute concentrations of EPA and AD dementia and a borderline significant association between the relative proportion of EPA and AD dementia but non-significant findings for the relationships between the other percentage PUFA and dementia and AD dementia. The effect of the relative proportions of the investigated PUFA might have been attenuated by the proportions of other fatty acids. Thus, the present study reflects the aforementioned arguments that similar findings can be observed for absolute concentrations and percentage distribution of PUFA, but there might be a slight advantage for absolute concentrations.

### 4.3. Strengths and Limitations

An important strength of our study is that we were able to measure absolute concentrations in addition to relative proportion of PUFA in serum phospholipids in the oldest old. Furthermore, our study has a prospective longitudinal study design in which we could investigate the predictive value of n-3 and n-6 PUFAs in the oldest old. In addition, we were able to adjust for a wide range of important confounders, including cognitive decline, to account for reverse causation. However, we recognize that reverse causation might still be present. It might be that increased inflammatory processes before disease onset have led to decreased concentrations of the n-3 PUFA in the circulation due to their function as anti-inflammatory agents [65]. Indeed, lower concentrations of plasma n-3 PUFA measures have been reported in cognitively impaired cases versus controls [66]. Furthermore, our study has an observational study design, which implies that a causal relationship cannot be established. Furthermore, we assessed serum phospholipids, which are sourced from a combination of exogenous and endogenous sources [67]. Herein, serum contains a mixture of phospholipids, triglycerides, and cholesteryl esters from lipoproteins and free fatty acids released from adipose tissue stores [67]. Thus, serum offers the advantage of sourcing fatty acids from multiple fatty acid depots [67]. By doing this, serum fatty acids may overcome limitations of the specific lipid species imparted by their turnover rates and levels of physiological control [67]. However, we are aware that PUFA profiles of other tissues (e.g., adipose tissue) or blood cells (e.g., erythrocytes) reflect long-term fatty acid intake [68]. In AgeCoDe, we were restricted to serum because the multi-center study design precluded rapid freezing of samples. However, the results can thus be extrapolated to a public health setting, where general practitioners send serum samples to a laboratory and provide patients with advice, e.g., on nutrition. Moreover, although the proportions of fatty acids vary by indifferent lipid fractions, previous studies showed strong correlations between fatty acid compositions of serum phospholipids and erythrocytes [21,69]. The correlations between fatty acid composition of serum phospholipids and erythrocytes suggest that the fatty acid composition of serum phospholipids influences the fatty acid composition of erythrocytes. Furthermore, while we were able to adjust for multiple confounders, residual confounding might still be present. Finally, our study was conducted in a German population, which limits the generalizability of our results to other ethnicities. We therefore recommend other studies to examine the associations under study in other populations.

## 5. Conclusions

In conclusion, higher concentrations of EPA were associated with a lower incidence of AD dementia. In addition, we observed that higher concentrations of EPA were associated with a decreased risk for all-cause and AD dementia among APOE ε4 non-carriers but not among APOE ε4 carriers. Furthermore, a higher percentage distribution of EPA, DHA, and ALA was not associated with incidence all-cause and AD dementia. We recommend future studies to analyze both relative fatty acid profiles and absolute fatty acid concentrations. In addition, we encourage existing and new studies to prioritize their research on the oldest old and to investigate the interaction between PUFA and APOE ε4.

## Figures and Tables

**Table 1:** Participants baseline characteristics.

Characteristic	Study Sample
	*n* = 1264
Age at study baseline (y)	84 ± 3
Female (*n*, %)	812 (64.0)
BMI (kg/m^2^)	25.9 ± 3.8
APOE ε4 status (*n*, %)	240 (19.0)
Duration to develop dementia (y)	5 ± 2
Cognitive decline score	−0.1 ± 2.1
Serum parameters	
alpha-tocopherol (mg/L)	15.71 ± 6.36
Triglycerides (g/L)	1.18 (IQR: 0.93–1.39)
Total cholesterol (g/L)	2.21 ± 0.47
Education (*n*, %)	
Lower	734 (58.1)
Middle	375 (29.7)
High	155 (12.3)
Physical activity index score * (*n*, %)	
Low (0–1)	102 (8.1)
Middle (2)	853 (67.5)
High (3–5)	309 (24.4)
Smoking (*n*, %)	
Never	647 (51.2)
Past	542 (42.9)
Current	75 (5.9)
Lipid-lowering medication ^¥^ (*n*, %)	256 (20.3)

Based on imputed data. Values are means ± SD, numbers (valid percentages), or medians (interquartile range). Abbreviations: *n* = number of participants; y = years; mg/L = milligrams per liter; mg/dl = milligrams per deciliter; g/L = grams per liter. * The physical activity index includes six activities: bicycling, walking, swimming, gymnastics, chores/gardening, and a category of other physical leisure activities. Participants were given a score of 1 (i.e., once a week to each day = 1) or 0 (i.e., less than once a week or never = 0), which were summed up across the six activities to a total physical activity score (score: 0–6). ^¥^ Statins and fibrates use.

**Table 2:** Omega-3 and omega-6 fatty acids measured in serum phospholipids (*n* = 1264).

Fatty Acids	Fatty Acid Concentrations (µmol/L)	Fatty Acid Composition (% of Total Fatty Acids)	Spearman’s Correlations between Fatty Acid Concentration and Composition
**Omega-3 fatty acids**			
alpha-linolenic acid	10 ± 7	0.17 ± 0.08	0.830
Eicosapentaenoic acid	72 ± 50	1.08 ± 0.65	0.861
Docosahexaenoic acid	217 ± 105	3.77 ± 1.10	0.490
**Omega-6 fatty acids**			
Linoleic acid	990 ± 406	18.05 ± 2.90	0.615
Dihomo-γ-linolenic acid	153 ± 59	2.75 ± 0.66	0.688
Arachidonic acid	618 ± 239	9.14 ± 2.03	0.545

Values are means ± standard deviations. Abbreviations: *n* = number of participants, % = percentage.

**Table 3:** Longitudinal associations between concentrations of n-3 and n-6 fatty acids in serum phospholipids and incident all-cause dementia over a 7-year follow-up period (*n* = 1264).

Fatty acids	Model 1		Model 2	
	*HR* (95% CI)	*p*	*HR* (95% CI)	*p*
**Omega-3 fatty acids**				
alpha-Linolenic acid (SD)	0.91 (0.78; 1.06)	0.227	0.94 (0.81; 1.10)	0.427
Eicosapentaenoic acid (SD)	0.86 (0.74; 1.01)	0.057	0.88 (0.75; 1.02)	0.092
Docosahexaenoic acid (SD)	0.88 (0.77; 1.03)	0.112	0.92 (0.79; 1.07)	0.283
**Omega-6 fatty acids**				
Linoleic acid (SD)	1.02 (0.90; 1.17)	0.744	1.05 (0.93; 1.19)	0.415
Dihomo-γ-linolenic acid (SD)	0.99 (0.86; 1.13)	0.885	1.01 (0.88; 1.17)	0.891
Arachidonic acid (SD)	0.97 (0.85; 1.12)	0.698	0.96 (0.84; 1.10)	0.562

Abbreviations: DHA = docosahexaenoic acid; EPA= eicosapentaenoic acid; n-3 = omega-3 fatty acids; n-6 = omega-6 fatty acids; HR = hazard ratio. Model 1: age, gender, apolipoprotein E ε4 (*APOE* ε4), education. Model 2: Model 1 plus BMI, physical activity, smoking, cognitive decline, alpha-tocopherol, triglycerides, total cholesterol, and lipid-lowering medication (i.e., statins and fibrates). A *p*-value < 0.05 is considered to be statistical significant.

**Table 4:** Longitudinal associations between concentrations of n-3 and n-6 fatty acids in serum phospholipids and incident AD dementia over a 7-year follow-up period (*n* = 1221).

Fatty acids	Model 1		Model 2	
	*HR* (95% CI)	*p*	*HR* (95% CI)	*p*
**Omega-3 fatty acids**				
alpha-Linolenic acid (SD)	0.88 (0.73; 1.05)	0.148	0.91 (0.76; 1.09)	0.286
Eicosapentaenoic acid (SD)	0.75 (0.62; 0.92)	0.004	0.76 (0.63; 0.93)	0.008
Docosahexaenoic acid (SD)	0.84 (0.71; 0.99)	0.043	0.87 (0.74; 1.04)	0.123
**Omega-6 fatty acids**				
Linoleic acid (SD)	1.04 (0.91; 1.19)	0.547	1.07 (0.94; 1.20)	0.307
Dihomo-γ-linolenic acid (SD)	1.00 (0.87; 1.17)	0.959	1.02 (0.88; 1.20)	0.762
Arachidonic acid (SD)	0.96 (0.83; 1.12)	0.625	0.94 (0.81; 1.10)	0.469

Abbreviations: AD = Alzheimer’s disease; DHA = docosahexaenoic acid; EPA = eicosapentaenoic acid; n-3 = omega-3 fatty acids; n-6 = omega-6 fatty acids. Model 1: age, gender, *APOE* ε4, education. Model 2: Model 1 plus BMI, physical activity, smoking, cognitive decline, alpha-tocopherol, triglycerides, total cholesterol, and lipid-lowering medication (i.e., statins and fibrates). A *p*-value < 0.05 is considered to be statistical significant.

**Table 5:** Longitudinal associations between percentage n-3 and n-6 fatty acids in serum phospholipids and incident all-cause dementia over a 7-year follow-up period (*n* = 1264).

Fatty acids	Model 1		Model 2	
	*HR* (95% CI)	*p*	*HR* (95% CI)	*p*
**Omega-3 fatty acids**				
alpha-Linolenic acid (SD)	0.92 (0.80; 1.05)	0.213	0.95 (0.82; 1.09)	0.420
Eicosapentaenoic acid (SD)	0.91 (0.79; 1.06)	0.216	0.93 (0.81; 1.08)	0.332
Docosahexaenoic acid (SD)	1.04 (0.91; 1.18)	0.566	1.08 (0.95; 1.23)	0.248
**Omega-6 fatty acids**				
Linoleic acid (SD)	1.04 (0.91; 1.18)	0.577	1.07 (0.94; 1.22)	0.311
Dihomo-γ-linolenic acid (SD)	1.03 (0.90; 1.18)	0.631	1.05 (0.91; 1.21)	0.490
Arachidonic acid (SD)	1.02 (0.90; 1.16)	0.739	0.98 (0.85; 1.13)	0.797

Abbreviations: DHA = docosahexaenoic acid; EPA= eicosapentaenoic acid; n-3 = omega-3 fatty acids; n-6 = omega-6 fatty acids. Model 1: age, gender, *APOE* ε4, education. Model 2: Model 1 plus BMI, physical activity, smoking, cognitive decline, alpha-tocopherol, triglyceridesα, total cholesterol, and lipid-lowering medication (i.e., statins and fibrates). A *p*-value < 0.05 is considered to be statistical significant.

**Table 6:** Longitudinal associations between percentage n-3 and n-6 fatty acids in serum phospholipids and incident AD dementia over a 7-year follow-up period (*n* = 1221).

Fatty acids	Model 1		Model 2	
	*HR* (95% CI)	*p*	*HR* (95% CI)	*p*
**Omega-3 fatty acids**				
alpha-Linolenic acid (SD)	0.90 (0.76; 1.05)	0.172	0.93 (0.79; 1.09)	0.345
Eicosapentaenoic acid (SD)	0.84 (0.70; 1.00)	0.051	0.86 (0.72; 1.03)	0.096
Docosahexaenoic acid (SD)	1.05 (0.91; 1.21)	0.516	1.11 (0.96; 1.28)	0.176
**Omega-6 fatty acids**				
Linoleic acid (SD)	1.08 (0.93; 1.25)	0.324	1.10 (0.96; 1.28)	0.175
Dihomo-γ-linolenic acid (SD)	1.07 (0.92; 1.24)	0.391	1.09 (0.94; 1.28)	0.264
Arachidonic acid (SD)	1.00 (0.88; 1.16)	0.989	0.96 (0.82; 1.12)	0.589

Abbreviations: AD = Alzheimer’s disease; DHA= docosahexaenoic acid; EPA = eicosapentaenoic acid; n-3 = omega-3 fatty acids; n-6 = omega-6 fatty acids. Model 1: age, gender, *APOE* ε4, education. Model 2: Model 1 plus BMI, physical activity, smoking, cognitive decline, alpha-tocopherol, triglycerides, total cholesterol, and lipid-lowering medication (i.e., statins and fibrates). A *p*-value < 0.05 is considered to be statistical significant.

## Data Availability

Data described in the manuscript, code book, and analytic code will be made available to qualified researchers upon reasonable request.

## Supplementary Materials

The following are available online at https://www.mdpi.com/2072-6643/13/2/461/s1, Table S1: Participants characteristics; Table S2: Association between concentrations of serum arachidonic acid and incident all-cause dementia over a 7-year follow-up period, stratified by APOE ε4 status; Table S3: Association between concentrations of serum alpha-linolenic acid and incident all-cause dementia over a 7-year follow-up period, stratified by APOE ε4 status; Table S4: Association between concentrations of serum EPA and incident all-cause dementia over a 7-year follow-up period, stratified by APOE ε4 status; Table S5: Association between concentrations of serum EPA and incident AD dementia over a 7-year follow-up period, stratified by APOE ε4 status; Table S6: Association between proportions of serum EPA and incident all-cause dementia over a 7-year follow-up period, stratified by APOE ε4 status; Table S7: Association between proportions of serum EPA and incident AD dementia over a 7-year follow-up period, stratified by APOE ε4 status; Table S8: Specification of the multiple imputation procedure.

## Abbreviations

AD: Alzheimer’s disease; AgeCoDe: German Study on Ageing, Cognition, and Dementia in Primary Care Patients; LA, linoleic acid; DGLA = dihomo-γ-linolenic acid; AA, arachidonic acid; ALA, alpha-linolenic acid; EPA, eicosapentaenoic acid; DHA, docosahexaenoic acid.
