# Supplementary material for: Eicosapentaenoic Acid Is Associated with Decreased Incidence of Alzheimer’s Dementia in the Oldest Old
Source: Nutrients. 2021 Jan 30;13(2):461. doi: 10.3390/nu13020461 (PMC7912244; doi:10.3390/nu13020461)
Supplement: Supplementary file 1 [file nutrients-13-00461-s001.pdf]

Online Supporting Material

Table S1. Participants baseline characteristics

Characteristic	Study sample<br>n=1,264
Age at baseline (y)	84 ± 3
Female (n, %)	812 (64.0)
BMI (kg/m^2)	25.9 ± 3.7
Missing values	29
APOE ε4 status (n, %)	230 (18.2)
Missing values	*41 (3.2)*
Duration to develop dementia (y)	5 ± 2
Cognitive decline score	-0.1 ± 2.1
Serum parameters	
alpha-tocopherol (mg/L)	15.7 ± 6.4
Missing values	*11*
Triglycerides (g/L)	1.11 (IQR: 0.87-1.48)
Missing values	*252*
Total cholesterol (g/L)	2.21 ± 0.48
Missing values	*29*
Education (n, %)	
Lower	734 (58.1)
Middle	375 (29.7)
High	155 (12.3)
Physical activity index score* (n, %)	
Low (0-1)	100 (7.9)
Middle (2)	836 (66.1)
High (3-5)	302 (23.9)
Missing values	*26 (2.1)*
Smoking (n, %)	
Never	647 (51.2)
Past	542 (42.9)
Current	75 (5.9)
Lipid lowering medication[¥] (n, %)	256 (20.3)

Values are means ± SD, numbers (valid percentages), or medians (interquartile range).
Abbreviations: g/L= grams per Liter; mg/L= milligrams per liter; n= number of participants; y=years. *The physical activity index includes six activities: bicycling, walking, swimming, gymnastics, chores/gardening, and a category of other physical leisure activities. Participants were given a score of 1 (i.e. once a week to each day = 1) or 0 (i.e. less than once a week or never= 0), which were summed up across the six activities to a total physical activity score (score: 0-6). [¥]Statins and fibrates use.

Table S2. Association between concentrations of serum arachidonic acid and incident all-cause dementia over a 7-year follow-up, stratified by *APOE* ε4 status

	***APOE* ε4 non-carriers** (n=1,024)	
**Fatty acid**	**All-cause dementia**	
	Model 1 *HR* (95%CI)	Model 2 *HR* (95%CI)
Arachidonic acid (SD)	0.91 (0.76; 1.07)	0.88 (0.74; 1.05)
P-value	*0.246*	*0.153*

	***APOE* ε4 carriers** (n=240)	
**Fatty acid**	**All-cause dementia**	
	Model 1 *HR* (95%CI)	Model 2 *HR* (95%CI)
Arachidonic acid (SD)	1.17 (0.91; 1.49)	1.18 (0.91; 1.53)
P-value	*0.229*	*0.212*

Based on imputed data. P for interaction=0.078.

Abbreviations: *APOE* ε4= apolipoprotein E ε4; CI= confidence interval.

Model 1: age, sex and education.

Model 2: model 1 plus BMI, physical activity, smoking, cognitive decline, alpha- tocopherol, triglycerides, total cholesterol and lipid lowering medication (i.e. statins and fibrates). A P-value <0.05 is considered to be statistical significant.

Table S3. Association between concentrations of serum alpha-Linolenic acid and incident all-cause dementia over a 7-year follow-up, stratified by *APOE* ε4 status

	***APOE* ε4 non-carriers** (n=1,024)	
**Fatty acid**	**All-cause dementia**	
	Model 1 *HR* (95%CI)	Model 2 *HR* (95%CI)
alpha-Linolenic acid (SD)	0.86 (0.71; 1.05)	0.90 (0.75; 1.09)
P-value	*0.138*	*0.293*

	***APOE* ε4 carriers** (n=240)	
**Fatty acid**	**All-cause dementia**	
	Model 1 *HR* (95%CI)	Model 2 *HR* (95%CI)
alpha-Linolenic acid (SD)	1.00 (0.76; 1.32)	0.96 (0.71; 1.29)
P-value	*0.978*	*0.764*

Based on imputed data. P for interaction=0.052.
Abbreviations: *APOE* ε4= apolipoprotein E ε4; CI= confidence interval.
Model 1: age, sex and education.
Model 2: model 1 plus BMI, physical activity, smoking, cognitive decline, alpha- tocopherol, triglycerides, total cholesterol and lipid lowering medication (i.e. statins and fibrates). A P-value <0.05 is considered to be statistical significant.

Table S4. Association between concentrations of serum EPA and incident all-cause dementia over a 7-year follow-up, stratified by *APOE* ε4 status

	***APOE* ε4 non-carriers** (n=1,024)	
**Fatty acid**	**All-cause dementia**	
	Model 1 *HR* (95%CI)	Model 2 *HR* (95%CI)
Eicosapentaenoic acid (SD)	0.75 (0.61; 0.92)	0.76 (0.61; 0.94)
P-value	***0.006***	***0.010***

	***APOE* ε4 carriers** (n=240)	
**Fatty acid**	**All-cause dementia**	
	Model 1 *HR* (95%CI)	Model 2 *HR* (95%CI)
Eicosapentaenoic acid (SD)	1.08 (0.87; 1.33)	1.06 (0.86; 1.31)
P-value	*0.497*	*0.592*

Based on imputed data. P for interaction=0.004.
Abbreviations: *APOE* ε4= apolipoprotein E ε4; CI= confidence interval; EPA= Eicosapentaenoic acid.
Model 1: age, sex and education.
Model 2: model 1 plus BMI, physical activity, smoking, cognitive decline, alpha- tocopherol, triglycerides, total cholesterol and lipid lowering medication (i.e. statins and fibrates). A P-value <0.05 is considered to be statistical significant.

Table S5. Association between concentrations of serum EPA and incident AD dementia over a 7-year follow-up, stratified by *APOE* ε4 status

	***APOE* ε4 non-carriers** (n=994)	
**Fatty acid**	**AD dementia**	
	Model 1 *HR* (95%CI)	Model 2 *HR* (95%CI)
Eicosapentaenoic acid (SD)	0.66 (0.51; 0.84)	0.66 (0.51; 0.85)
P-value	***0.001***	***0.001***
	***APOE* ε4 carriers** (n=227)	
**Fatty acid**	**AD dementia**	
	Model 1 *HR* (95%CI)	Model 2 *HR* (95%CI)
Eicosapentaenoic acid (SD)	0.98 (0.74; 1.29)	0.98 (0.72; 1.32)
P-value	*0.888*	*0.868*

Based on imputed data. P for interaction=0.021.
Abbreviations: AD= Alzheimer's disease; *APOE* ε4= apolipoprotein E ε4; CI= confidence interval; EPA= eicosapentaenoic acid.
Model 1: age, sex and education.
Model 2: model 1 plus BMI, physical activity, smoking, cognitive decline, alpha- tocopherol, triglycerides, total cholesterol and lipid lowering medication (i.e. statins and fibrates). A P-value <0.05 is considered to be statistical significant.

Table S6. Association between proportions of serum EPA and incident all-cause dementia over a 7-year follow-up, stratified by *APOE* ε4 status

	***APOE* ε4 non carriers** (n=1,024)	
**Fatty acid**	**All-cause dementia**	
	Model 1 *HR* (95%CI)	Model 2 *HR* (95%CI)
Eicosapentaenoic acid (SD)	0.86 (0.71; 1.02)	0.87 (0.73; 1.04)
P-value	*0.086*	*0.128*

	***APOE* ε4 carriers** (n=240)	
**Fatty acid**	**All-cause dementia**	
	Model 1 *HR* (95%CI)	Model 2 *HR* (95%CI)
Eicosapentaenoic acid (SD)	1.07 (0.84; 1.35)	1.08 (0.85; 1.37)
P-value	*0.592*	*0.521*

Based on imputed data. P for interaction=0.061.
Abbreviations: *APOE* ε4= apolipoprotein E ε4; CI= confidence interval; EPA= eicosapentaenoic acid.
Model 1: age, sex and education.
Model 2: model 1 plus BMI, physical activity, smoking, cognitive decline, alpha- tocopherol, triglycerides, total cholesterol and lipid lowering medication (i.e. statins and fibrates). A P-value <0.05 is considered to be statistical significant.

Table S7. Association between proportions of serum EPA and incident AD dementia over a 7-year follow-up, stratified by *APOE* ε4 status

	***APOE* ε4 non-carriers** (n=994)	
**Fatty acid**	**AD dementia**	
	Model 1 *HR* (95%CI)	Model 2 *HR* (95%CI)
Eicosapentaenoic acid (SD)	0.79 (0.63; 0.97)	0.80 (0.65; 0.99)
P-value	***0.028***	***0.042***

	***APOE* ε4 carriers** (n=227)	
**Fatty acid**	**AD dementia**	
	Model 1 *HR* (95%CI)	Model 2 *HR* (95%CI)
Eicosapentaenoic acid (SD)	0.98 (0.73; 1.32)	1.03 (0.76; 1.41)
P-value	*0.912*	*0.868*

Based on imputed data. P for interaction=0.067.
Abbreviations: AD= Alzheimer's disease; *APOE* ε4= apolipoprotein E ε4; CI= confidence interval; EPA= eicosapentaenoic acid;
Model 1: age, sex and education.
Model 2: model 1 plus BMI, physical activity, smoking, cognitive decline, alpha- tocopherol, triglycerides, total cholesterol and lipid lowering medication (i.e. statins and fibrates). A P-value <0.05 is considered to be statistical significant.

Table S8. Specification of the multiple imputation procedure

Software	IBM SPSS Statistics for Windows (Release 23)
Imputation method	Fully conditional specification (Markov chain Monte Carlo method)
Key settings	Maximum iterations: 20
Imputed data sets	10
Variables included in the imputation procedure (imputed or used as predictors of missing	Linoleic acid (%); Linoleic acid (μMol/L); DGLA (%); DGLA (μMol/L); Arachidonic acid (%); Arachidonic acid (μMol/L); alpha-Linoleic acid (%); alpha-Linoleic acid (μMol/L); EPA (%); EPA (μMol/L); DHA (%); DHA (μMol/L); Follow up 3 aggregated variable: Healthy controls, MCI, dementia variable; Incident AD dementia FU8; incident all-cause dementia FU8; vascular dementia FU8; sex; age at FU3; *APOE* ε4; education; body weight at FU3 (kg); height at FU3 (cm); smoking; physical activity at FU3; CERAD delate recall baseline score; CERAD delate recall FU3 score; cholesterol (mg/dl); triglycerides (mg/dl); lipid lowering medication
Additionally added predictive variables to increase plausibility of missing at random	Body weight at FU4 (kg); height at FU4 (cm); HDL cholesterol FU3 (mg/dl), LDL cholesterol (mg/dl) FU3; HbA1c; fasting state at blood sampling; systolic blood pressure (mmHg) FU3; diastolic blood pressure (mmHg) FU3; depression FU3; coronary heart disease FU3; myocard infarct FU3; heart failure FU3; TIA FU3; skull or brain trauma FU3; hyperlipidemia and hyper cholesterolemia FU3; ability to conduct chorus around the house FU3; hypertension FU3; diabetes mellitus (unspecified) FU3; kidney failure FU3; elevated blood pressure FU3; medication use FU2 and FU3
Not normally distributed variables were treated with:	Predictive mean matching
Binary/categorical variables were treated with:	Logistic regression models

Abbreviations: AD= Alzheimer's disease; *APOE* ε4; Apolipoprotein E ε4; CERAD= Consortium to Establish a Registry for Alzheimer's Disease; DGLA= Dihomo-γ-linolenic acid; DHA= docosahexaenoic acid; EPA= Eicosapentaenoic acid; FU= follow up visit; HbA1c= Hemoglobine A1c; HDL= high density lipoprotein; LDL= low density lipoprotein; TIA= trancient ischemic attack.
